# Infection by the Protozoan Parasite *Toxoplasma gondii* Inhibits Host MNK1/2-eIF4E Axis to Promote Its Survival

**DOI:** 10.3389/fcimb.2020.00488

**Published:** 2020-09-09

**Authors:** Louis-Philippe Leroux, Visnu Chaparro, Maritza Jaramillo

**Affiliations:** Institut National de la Recherche Scientifique (INRS)-Centre Armand-Frappier Santé Biotechnologie (CAFSB), Laval, QC, Canada

**Keywords:** *Toxoplasma gondii*, MNK1/2, eIF4E phosphorylation, p38 MAPK, macrophages, IFNγ, inflammation

## Abstract

The obligate intracellular parasite *Toxoplasma gondii* reprograms host gene expression through multiple mechanisms that promote infection, including the up-regulation of mTOR-dependent host mRNA translation. In addition to the mTOR-4E-BP1/2 axis, MAPK-interacting kinases 1 and 2 (MNK1/2) control the activity of the mRNA cap-binding protein eIF4E. Herein, we show that *T. gondii* inhibits the phosphorylation of MNK1/2 and their downstream target eIF4E in murine and human macrophages. Exposure to soluble *T. gondii* antigens (STAg) failed to fully recapitulate this phenotype indicating the requirement of live infection. Treatment with okadaic acid, a potent phosphatase inhibitor, restored phosphorylation of MNK1/2 and eIF4E regardless of infection. *T. gondii* replication was higher in macrophages isolated from mice mutated at the residue where eIF4E is phosphorylated (eIF4E S209A knock-in) than in wild-type (WT) control cells despite no differences in infection rates. Similarly, parasitemia in the mesenteric lymph nodes and spleen, as well as brain cyst burden were significantly augmented in infected eIF4E S209A knock-in mice compared to their WT counterparts. Of note, mutant mice were more susceptible to acute toxoplasmosis and displayed exacerbated levels of IFNγ. In all, these data suggest that the MNK1/2-eIF4E axis is required to control *T. gondii* infection and that its inactivation represents a strategy exploited by the parasite to promote its survival.

## Introduction

*Toxoplasma gondii* (*T. gondii*), the etiologic agent of toxoplasmosis, is an intracellular protozoan parasite that infects a wide variety of vertebrate hosts, including humans and mice (Innes et al., 2019). It is estimated that about 30–50% of the world population is seropositive for *T. gondii* (Montazeri et al., 2017). Toxoplasmosis is generally asymptomatic yet reactivation of encysted parasites can lead to life-threatening consequences in immuno-compromised individuals (Luft and Remington, 1992), or cause abortions or birth defects if contracted during pregnancy (Montoya and Remington, 2008). *T. gondii* is able to invade any nucleated cell and usurps host cell organelles and nutrients in order to replicate within its parasitophorous vacuole (Clough and Frickel, 2017). The parasite targets signaling pathways and host gene expression to subvert immune responses and establish a favorable environment (Hakimi et al., 2017; Blume and Seeber, 2018; Delgado Betancourt et al., 2019). Among the different strategies employed by the parasite, it was shown that *T. gondii* is able to fine-tune host gene expression post-transcriptionally in part through perturbations in translational efficiency of host mRNAs (Leroux et al., 2018).

Translational control enables cells to rapidly adapt their proteome to respond to stress or other metabolic cues without *de novo* mRNA synthesis (Gebauer and Hentze, 2004; Sonenberg and Hinnebusch, 2009). Changes in translational efficiency represent a fundamental mechanism in normal biological processes including cell differentiation, growth, metabolism, and proliferation (Gebauer and Hentze, 2004; Hershey et al., 2012). Translational control is also required for balanced immune functions (Piccirillo et al., 2014) and is observed during infectious diseases (Alain et al., 2010; Mohr and Sonenberg, 2012; Walsh et al., 2013; Nehdi et al., 2014; Leroux et al., 2018; Hoang et al., 2019; Chaparro et al., 2020). In eukaryotic cells, translational efficiency is mainly regulated at the initiation step during which ribosomes are recruited to the mRNA, a process facilitated by recognition of the mRNA 5′-m^7^G-cap structure by eukaryotic initiation factor 4E (eIF4E), which, together with scaffold protein eIF4G and RNA helicase eIF4A, form the eIF4F complex (Jackson et al., 2010). Assembly of the eIF4F complex is precluded by eIF4E-binding proteins (4E-BPs), which block eIF4E:eIF4G interaction and eIF4F formation (Pause et al., 1994; Lin and Lawrence, 1996). Hyper-phosphorylation of 4E-BPs by mechanistic target of rapamycin (mTOR) complex 1 (mTORC1) lowers 4E-BPs' affinity to eIF4E, thus favoring eIF4E:eIF4G interaction and initiation of translation (Gingras et al., 2001). Phosphorylation of eIF4E at residue S209 is an additional regulatory mechanism of translation initiation (Pelletier et al., 2015), and is mediated by MAP kinase-interacting serine/threonine-protein kinase 1 and 2 (MNK1/2) (Ueda et al., 2004). MNK1/2 are phosphorylated by upstream kinases, specifically p38 MAPK and ERK1/2, following various stimuli (ex: growth factors, cytokines, etc.) (Waskiewicz et al., 1997).

Some studies have reported an increase in translation upon eIF4E phosphorylation (Furic et al., 2010; Robichaud et al., 2015). In contrast, others have suggested that phosphorylation of eIF4E lowers its affinity for the mRNA cap structure (Scheper et al., 2002; Zuberek et al., 2003, 2004; Slepenkov et al., 2006). These seemingly contradictory observations could be reconciled by the possibility that reduced cap affinity favors eIF4E recycling and thus increases translation initiation rates (Scheper and Proud, 2002). Translation of transcripts with highly structured 5′ UTRs is facilitated through eIF4E activity, and RNA regulons controlling cell proliferation and survival are tightly regulated themselves by eIF4E (Volpon et al., 2019). In addition to its role in translation initiation, eIF4E carries out other functions including mRNA nuclear export, stability, and sequestration (Volpon et al., 2019). However, aberrant eIF4E activity is a determining factor in the development of various pathologies. Dysregulated MNK1/2 activity as well as elevated phosphorylated and total levels of eIF4E have been shown to promote oncogenesis and tumor growth (Proud, 2015). Phosphorylation of eIF4E was reported to increase translational efficiency of the mRNA encoding the NF-κB inhibitor IκBα. Hence, mice mutated at the residue where eIF4E is phosphorylated (S209A) were less susceptible to viral infections by virtue of enhanced NF-κB activity and type I interferon production (Herdy et al., 2012). In macrophages, efficient translation of HES-1 (Su et al., 2015), a transcriptional repressor of inflammatory genes, and IRF8 (Xu et al., 2012), a transcription factor that promotes M1 polarization, was shown to require MNK-mediated phosphorylation of eIF4E.

Modulation of eIF4E phosphorylation has been associated with enhanced viral replication (Kleijn et al., 1996; Walsh and Mohr, 2004). However, the role of the MNK1/2-eIF4E axis during infections by protozoan parasites has yet to be investigated. Here, we report that *T. gondii* reduces MNK1/2 and eIF4E phosphorylation levels and disrupts upstream signaling in infected macrophages. Importantly, we demonstrate that genetic ablation of eIF4E phosphorylation dramatically increases parasite replication *in vitro* as well as parasitemia and host susceptibility in an experimental toxoplasmosis model. These results highlight a central role for the MNK1/2-eIF4E axis in mitigating disease outcome during *T. gondii* infection.

## Materials and Methods

### Reagents

Culture media and supplements were purchased from Wisent; okadaic acid (*Prorocentrum* sp.) and phorbol-12-myristate-13-acetate (PMA) were acquired from Calbiochem; CellTracker Green (CMFDA) and DAPI were purchased from Invitrogen; Zombie Violet was supplied by BioLegend; resazurin sodium salt was acquired through Alfa Aesar; High Pure PCR Template Preparation Kit, and cOmplete EDTA-free protease inhibitor and PhosSTOP phosphatase inhibitor tablets were purchased from Roche; antibodies were acquired from Cell Signaling Technologies, R&D Systems, Sigma-Aldrich, and BD Biosciences.

### Differentiation of Murine Bone Marrow-Derived Macrophages

Bone marrow-derived macrophages (BMDMs) were generated from 6 to 8 week-old female C57BL/6 mice (Jackson Laboratory), as previously described (Leroux et al., 2018; Zakaria et al., 2018; Chaparro et al., 2020). Briefly, marrow was extracted from bones of the hind legs, red blood cells were lysed, and progenitor cells were resuspended in BMDM culture medium supplemented with 15% L929 fibroblast-conditioned culture medium (LCCM). Non-adherent cells were collected the following day and were cultured for 7 days in BMDM culture medium supplemented with 30% LCCM with fresh medium replenishment at day 3 of incubation.

### THP-1 Culture and Differentiation

The human monocytic cell line THP-1 (ATCC TIB-202) was maintained in suspension (DMEM, 10% heat-inactivated FBS, 2 mM L-glutamate, 1 mM sodium pyruvate, 100 U/mL penicillin, 100 μg/mL streptomycin, 20 mM HEPES, 55 μM β-mercaptoethanol). Cells were differentiated into macrophages by adding 20 ng/mL PMA for 24 h. The following day, spent medium was removed and fresh medium without PMA was added, and cells were allowed to rest for another 24 h prior to infection.

### Parasite Maintenance and Harvest

*T. gondii* cultures (RH and ME49 strains) were maintained by serial passages in Vero cells, as previously described (Leroux et al., 2018). For experimental infections, freshly egressed tachyzoites were harvested from Vero cultures, pelleted by centrifugation (1,300 × *g*, 7 min, 4°C), resuspended in ice-cold PBS (pH 7.2–7.4), and passed through a syringe fitted with a 27 G needle. Large cellular debris and intact host cells were pelleted by low-speed centrifugation (200 × *g*, 3 min, 4°C), and the supernatant containing parasites was filtered with a 3 μm-polycarbonate filter (Millipore). Tachyzoites were then washed twice in PBS and finally resuspended in the appropriate culture medium, according to the experiment.

### Soluble *T. gondii* Antigens (STAg)

STAg were prepared from freshly egressed tachyzoites, as previously described (Leroux et al., 2015a). Briefly, parasites were resuspended in ice-cold PBS, subjected to three 5-min cycles of freezing/thawing using liquid nitrogen and a 37°C water bath, then sonicated on ice for 5 min (1 s on/off pulses, 30% duty cycle) using a Sonic Dismembrator FB505 (ThermoFisher). Lysates were cleared by centrifugation (21,000 × *g*, 15 min, 4°C), and soluble material containing STAg was used for downstream experiments.

### Infection and Treatments of BMDM and THP-1 Cultures

Macrophages were plated 1 day before infection and allowed to adhere O/N at 37°C, 5% CO_2_. Cultures were serum-starved for 2 h and then inoculated with *T. gondii* (MOI 6:1; unless otherwise specified), treated with 50 μg/mL STAg (where applicable), or left uninfected in fresh medium with 1% FBS. Any remaining extracellular parasites were rinsed away with warm PBS (pH 7.2–7.4) 1 h following inoculation, and fresh medium was added. Cells were treated with 10 nM okadaic acid or DMSO 1 h after infection (where applicable).

### Western Blot Analysis

Cells were lysed in RIPA buffer supplemented with protease and phosphatase inhibitors, and samples were prepared for western blotting as described (Leroux et al., 2018; William et al., 2019). Primary antibodies anti-phospho-p38 (T180/Y182; #9216), anti-p38 (#8690), anti-phospho-ERK1/2 (T202/Y204; #9106), anti-ERK1/2 (#9102), anti-phospho-MNK1/2 (T197/202; #2111), anti-MNK1/2 (#2195), anti-phospho-eIF4E (S209; #9741), and anti-β-actin (#3700) were purchased from Cell Signaling Technologies; anti-eIF4E (#610269) was obtained from BD Biosciences; and anti-*T. gondii* profilin (#AF3860) was acquired from R&D Systems. Horseradish peroxidase (HRP)-linked goat anti-rabbit (#A0545) and goat anti-mouse IgG (#A4416) secondary antibodies were purchased from Sigma-Aldrich, and rabbit anti-goat (#HAF017) was acquired from R&D Systems. Densitometric analyses were performed with FIJI software.

### Experimental Toxoplasmosis

Tachyzoites were harvested as described above and resuspended in sterile PBS. WT and eIF4E S209A KI mice in the C57BL/6 background (Furic et al., 2010) were infected intraperitoneally with either 10^2^ RH or 10^3^ ME49 *T. gondii*, or mock infected with PBS. Serum, mesenteric lymph nodes (MLN), and spleens were collected 8 days post-infection (acute), while brains were harvested after 21 days (chronic) for downstream analyses. Mouse health status was monitored up to 21 days post-infection. At least 5 mice per genotype were monitored in each infection trial.

### Measurement of *in vitro* Parasite Replication, *in vivo* Parasitemia, and Cyst Burden by qPCR

*In vitro* parasite replication was evaluated by epifluorescence microscopy. Briefly, infected BMDM cultures were fixed at the indicated times with PBS with 3.7% PFA (15 min, RT). Cells were permeabilized with PBS with 0.2% Triton X-100 (5 min, RT), stained with DAPI (5 min, RT), then mounted onto slides. The number of parasites in at least 50 vacuoles in different fields for each genotype and time point was counted by microscopy using a 60X oil-immersion objective. The observer was blinded as to which sample was being evaluated to avoid bias during enumeration of the parasites. *In vivo* parasitemia and cyst burden were quantified by amplification of the *T. gondii B1* gene, as previously described (Leroux et al., 2015b, 2018). Genomic DNA (gDNA) was extracted from MLN, spleen, and brain tissues using High Pure PCR Template Preparation Kit (Roche) as per manufacturer's guidelines. *T. gondii B1* gene was amplified by qPCR using the PowerUP SYBR Green PCR Master Mix (Applied Biosystems) with the following primers: forward (5′-TCCCCTCTGCTGGCGAAAAGT-3′) and reverse (5′-AGCGTTCGTGGTCAACTATCGATTG-3′) (Integrated DNA Technologies). Reaction was carried out in a QuantStudio 3 Real-Time PCR System (Applied Biosciences). Values were normalized using the mouse β*-actin* gene amplified with forward (5′-CACCCACACTGTGCCCATCTACGA-3′) and reverse (5′- CAGCGGAACCGCTCATTGCCAATGG-3′) primers. Analysis was carried out by relative quantification using the Comparative C_t_ method (2^−ΔΔCt^) (Livak and Schmittgen, 2001).

### ELISA

Serum from acutely infected mice and mock-injected control mice was collected 8 days post-infection. IFNγ levels were measured by sandwich ELISA using a Mouse IFN-γ ELISA MAX Deluxe kit (Biolegend; #430804).

### Statistical Analyses

Where applicable, data are presented as mean [SD]. Statistical significance was determined using unpaired *T*-test followed by Welch's correction or paired *T*-test (for *in vitro* replication assay); calculations were performed using Prism Software (GraphPad). For survival curves, log-rank test (Mantel-Cox) was used to determine significance. Differences were considered significant when ^*^P < 0.05, ^**^P < 0.01, ^***^P < 0.001.

## Results

### *T. gondii* Inhibits Host MNK1/2 and eIF4E Phosphorylation and Disrupts Upstream Signaling in Infected Macrophages

**Figure 1:**
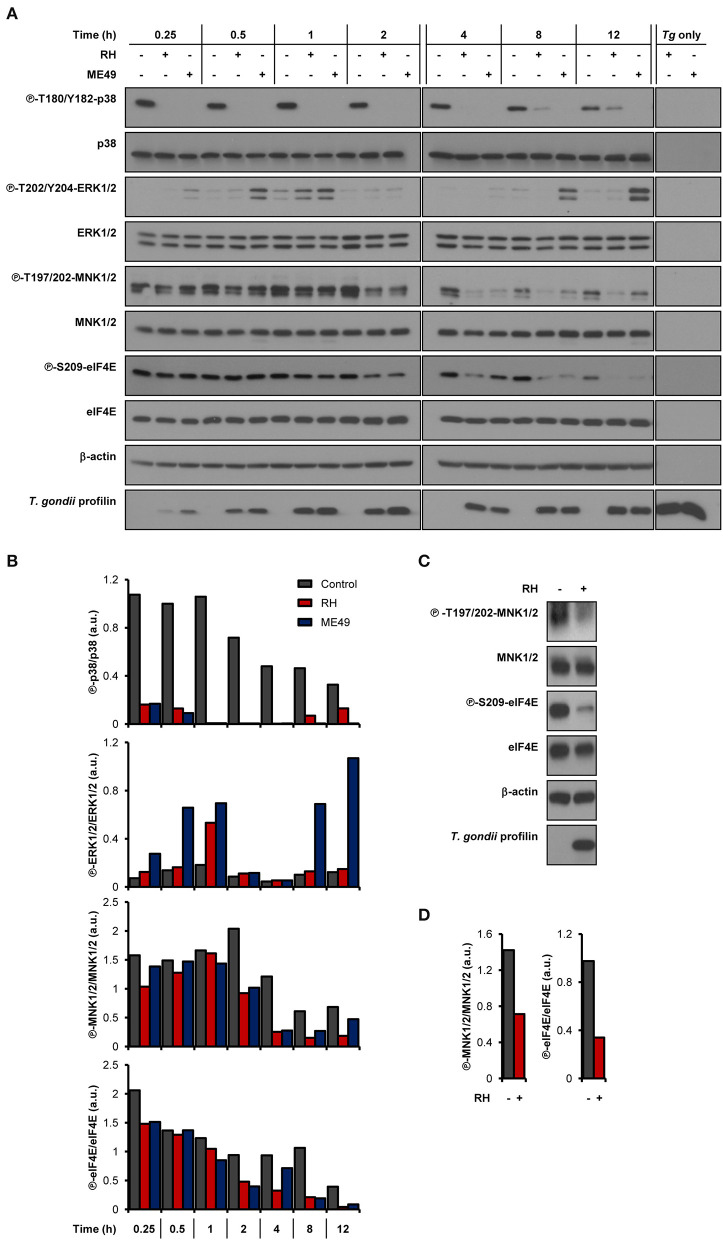
*T. gondii* represses MNK1/2-eIF4E signaling pathway in macrophages. **(A,B)** BMDM cultures were infected with either RH or ME49 for the indicated times or left uninfected. **(C,D)** PMA-differentiated THP-1 cells were infected with RH strain for 8 h or left uninfected. **(A,C)** Phosphorylation and expression levels of indicated proteins were monitored by western blotting. Total amounts of β-actin were used as a loading control and an antibody against *T. gondii* profilin-like protein was employed to assess the infection of the BMDM cultures. Total protein extracts from extracellular tachyzoites (RH and ME49) (*Tg* only) were used to control for any cross-reactivity of the antibodies against *T. gondii* proteins. **(B,D)** Densitometric analysis of the phosphorylation status of indicated proteins in uninfected control cultures and infected cells using the FIJI software. Data and data analyses are representative of at least two biological replicates.

As we and others have previously demonstrated, infection by *T. gondii* increases host mTOR signaling (Wang et al., 2010; Al-Bajalan et al., 2017) and mTOR-dependent mRNA translation (Leroux et al., 2018). In addition to the mTOR-4E-BP1/2 axis, MAPK-interacting kinases 1 and 2 (MNK1/2) control the activity of the mRNA cap-binding protein eIF4E (Proud, 2015). We therefore sought to determine if the cap-binding initiation factor eIF4E and its upstream signaling intermediates were activated upon infection by monitoring their phosphorylation status. Of note, parasite extracts (i.e., devoid of any host cell; “*Tg* only”) were probed in parallel to rule out the possibility that any observed changes in signaling were due to cross-reactivity of the antibodies against *T. gondii* proteins. We first assayed the phosphorylation status of the upstream kinases of MNK1/2, specifically p38 MAPK and ERK1/2 (Waskiewicz et al., 1997). Phosphorylation of p38 MAPK (T180/Y182) was severely compromised in infected BMDMs by both parasite strains (Figures 1A,B). As for ERK1/2, phosphorylation at residues T202/Y204 gradually increased over time in cells infected by ME49 only, revealing a strain-dependent modulation. Regardless of ERK1/2 activation, phosphorylation of MNK1/2 (T197/202) was reduced in BMDMs infected with either strain compared to uninfected control cells. Consistently, phosphorylation levels of eIF4E (S209) were readily abrogated and remained as such in infected BMDM cultures. The induction of ERK1/2 phosphorylation by ME49, and the inhibition of MNK1/2 and eIF4E phosphorylation by both RH and ME49 followed an MOI-dependent trend whereby the respective phenotypes were increasingly pronounced as the parasite-to-host ratio increased (Supplementary Figure 1). On the other hand, an MOI of 1:1 was enough to lead to the inhibition of p38 phosphorylation by both *T. gondii* strains. In addition, MNK1/2 and eIF4E phosphorylation levels were reduced in infected human THP-1 macrophages (Figures 1C,D). In summary, *T. gondii* infection inhibits the MNK1/2-eIF4E signaling axis in both murine and human macrophages.

### Live Parasites and Phosphatase Activity Are Required for Inhibition of the MNK1/2-eIF4E Axis During *T. gondii* Infection in Macrophages

**Figure 2:**
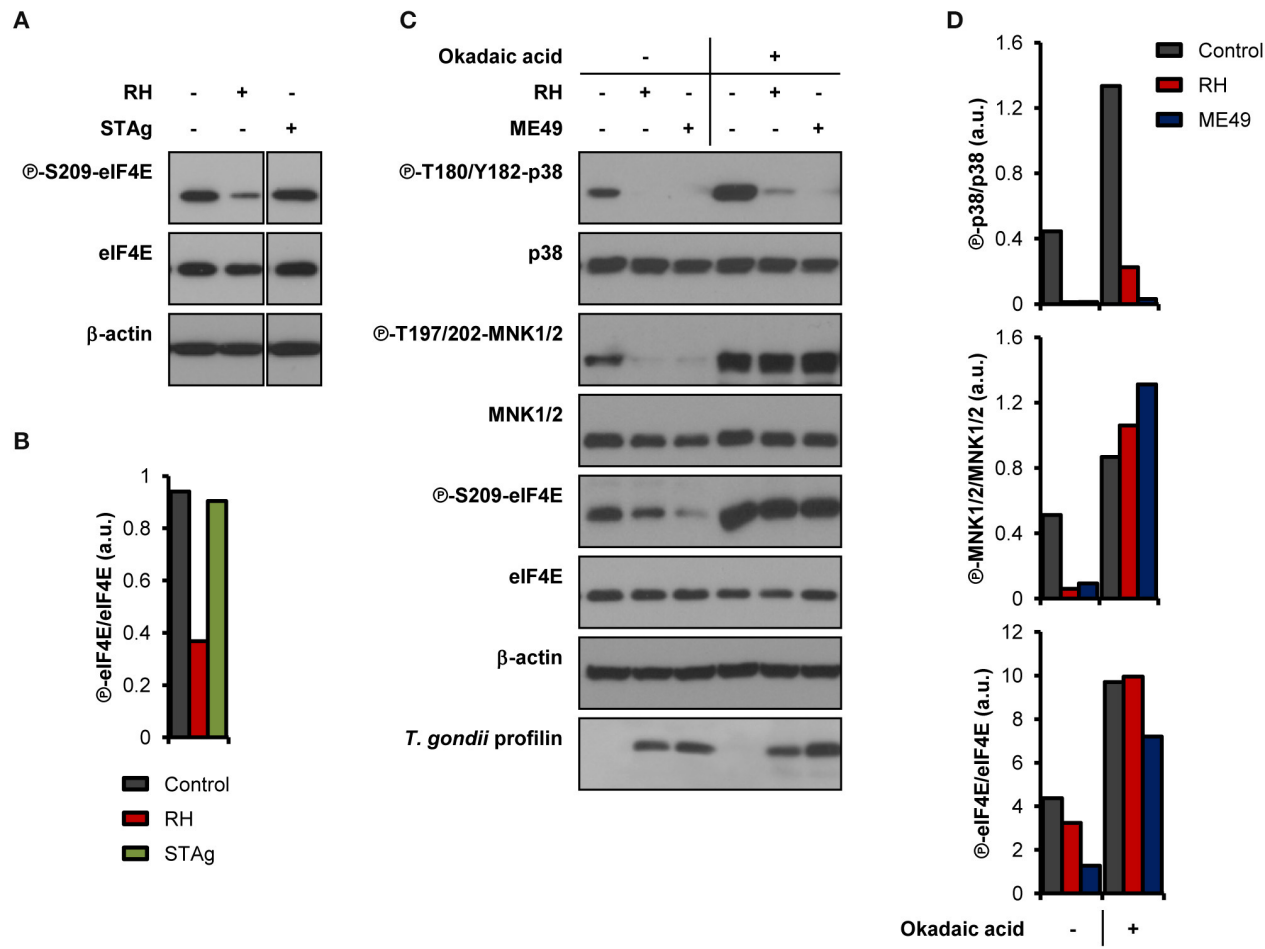
*T. gondii*-derived soluble antigens do not fully recapitulate inhibitory effects of live infection, while okadaic acid restores phosphorylation levels of MNK1/2-eIF4E. BMDM cultures were infected with **(A,B)** RH strain or treated with 50 μg/mL of STAg, or **(C,D)** infected with either RH or ME49 strains then treated with DMSO (vehicle) or 10 nM okadaic acid for 8 h. In all cases, uninfected cultures were collected in parallel. **(A,C)** Phosphorylation and expression levels of indicated proteins were monitored by western blotting. Total amounts of β-actin were used as a loading control and an antibody against *T. gondii* profilin-like protein was employed to assess the infection of the BMDM cultures. **(B,D)** Densitometric analysis of the phosphorylation status of indicated proteins in uninfected control cultures and infected cells using the FIJI software. Data and data analyses are representative of two biological replicates.

We next sought to determine if live infection was required to disrupt the activation of the MNK1/2-eIF4E pathway. Unlike infection with live parasites, treatment of BMDM cultures with soluble *T. gondii* antigens (STAg) failed to inhibit eIF4E phosphorylation (Figures 2A,B). To begin deciphering the molecular mechanisms involved in the inhibition of MNK1/2-eIF4E phosphorylation, we first infected BMDM cultures and 1 h later treated them with 10 nM okadaic acid, a potent protein phosphatase type 1 and 2A inhibitor (Li et al., 2010). This approach helped avoid any effects of the inhibitor on the parasite's ability to infect host cells. Treatment with okadaic acid restored phosphorylation of MNK1/2 and eIF4E regardless of infection by *T. gondii* (Figures 2C,D). However, p38 phosphorylation levels remained markedly inhibited in infected cells suggesting that different mechanisms are responsible for the dephosphorylation of p38 and MNK1/2-eIF4E observed upon infection. Of note, treatment with 10 to 50 nM okadaic acid did not affect the viability of BMDM cultures and extracellular *T. gondii* parasites up to 12 and 24 h, respectively (Supplementary Figures 2A,B). Taking together, these results indicate a complex repression of the MNK1/2-eIF4E axis by *T. gondii* that is independent of p38 inactivation but implicates parasite- and/or host-derived phosphatases that remain to be identified.

### Parasite Replication Within eIF4E S209A KI BMDMs Is Increased While 4E KI Mice Are More Susceptible to Toxoplasmosis

**Figure 3:**
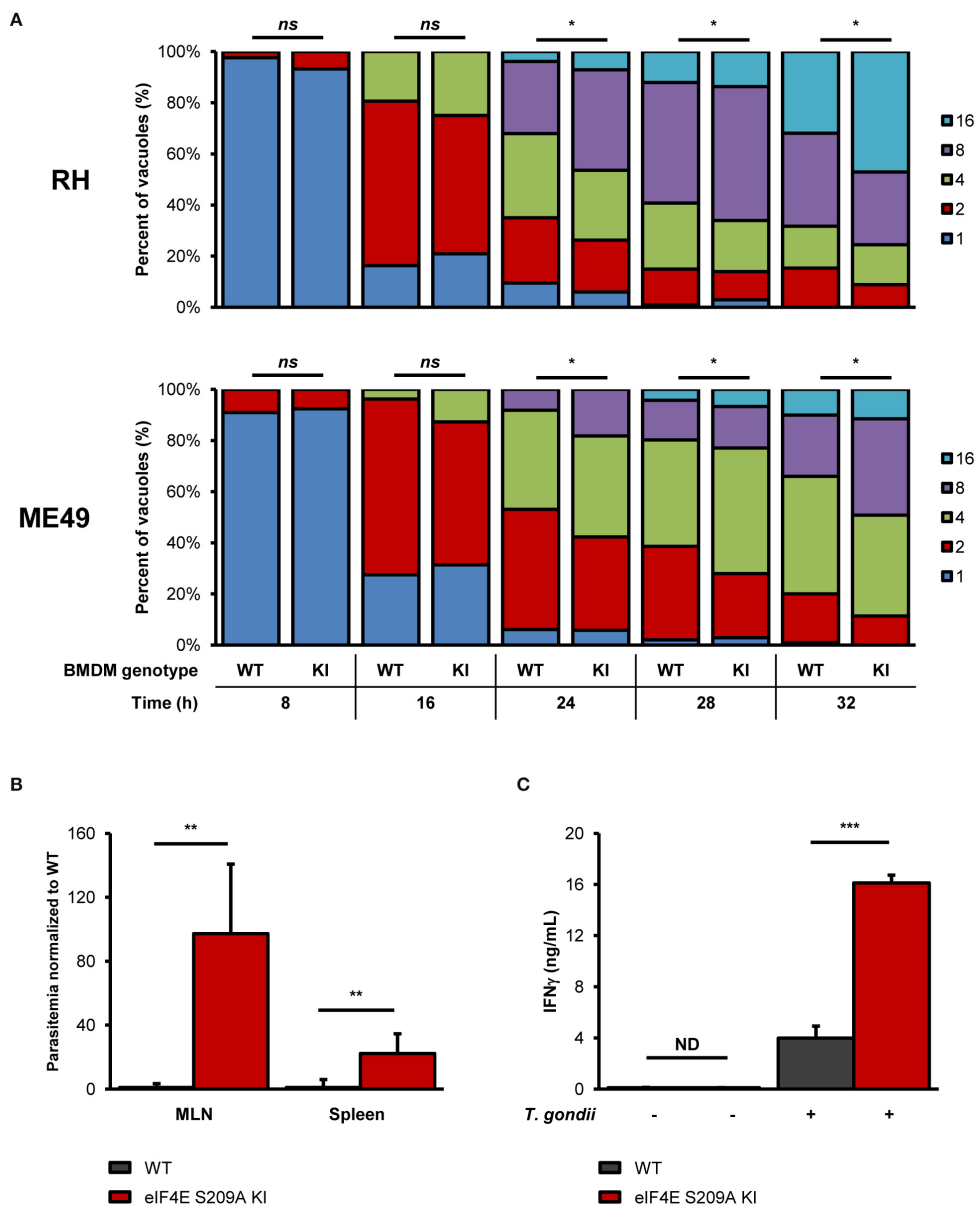
Genetic inhibition of eIF4E phosphorylation increases *in vitro* parasite replication, and exacerbates parasite burden and inflammation during experimental toxoplasmosis. **(A)** BMDM differentiated from WT or eIF4E S209A KI mice were infected with RH tachyzoites for the indicated time, fixed, stained with DAPI, and mounted onto slides. The number of parasites in at least 50 vacuoles in different fields for each genotype and time point was counted by epifluorescence microscopy. Data are representative of two biological replicates; “*ns*” refers to “not significant.” **(B,C)** WT and eIF4E S209A KI mice were inoculated intraperitoneally (IP) with 10^2^ RH tachyzoites or PBS (mock) and euthanized 8 days post-infection. **(B)** Parasitemia in the MLN and spleen was determined by qPCR by amplification of the *T. gondii B1* gene. Ct values were normalized to the mouse β*-actin* gene, and values are expressed as fold-change compared to WT mice. **(C)** Serum IFNγ levels were measured by sandwich ELISA. Results are presented as mean [SD] calculated from values obtained from infected mice (two independent experiments; at least 5 mice per group); all samples were analyzed in technical triplicates; “ND” refers to “not detected”. ^*^P < 0.05, ^**^P < 0.01, ^***^P < 0.001.

To begin evaluating the impact of eIF4E phosphorylation on *T. gondii* replication, we infected BMDM cultures generated from WT mice or mutated at the residue where eIF4E is phosphorylated (eIF4E S209A knock-in [KI]; 4E KI) (Furic et al., 2010). *In vitro* parasite replication was enhanced in eIF4E S209A KI BMDMs compared to WT cells as measured by microscopic analyses (Figure 3A). The average number of parasite per vacuole appeared to increase at a slightly higher rate for both RH and ME49 in the mutant host cells at 16 h post-infection, a phenotype was statistically significant at 24 h and on following infection. These data suggest that the inhibition of eIF4E phosphorylation in BMDMs represents a strategy that favors *T. gondii*. Importantly, the rate of infection (i.e., number of infected cells) did not differ between WT and 4E KI BMDM cultures (Supplementary Figures 3A,B). Furthermore, up-regulation of ERK1/2 phosphorylation, and inhibition of p38 and MNK1/2 phosphorylation by *T. gondii* were similar in WT and mutant macrophages (Supplementary Figures 3C,D).

**Figure 4:**
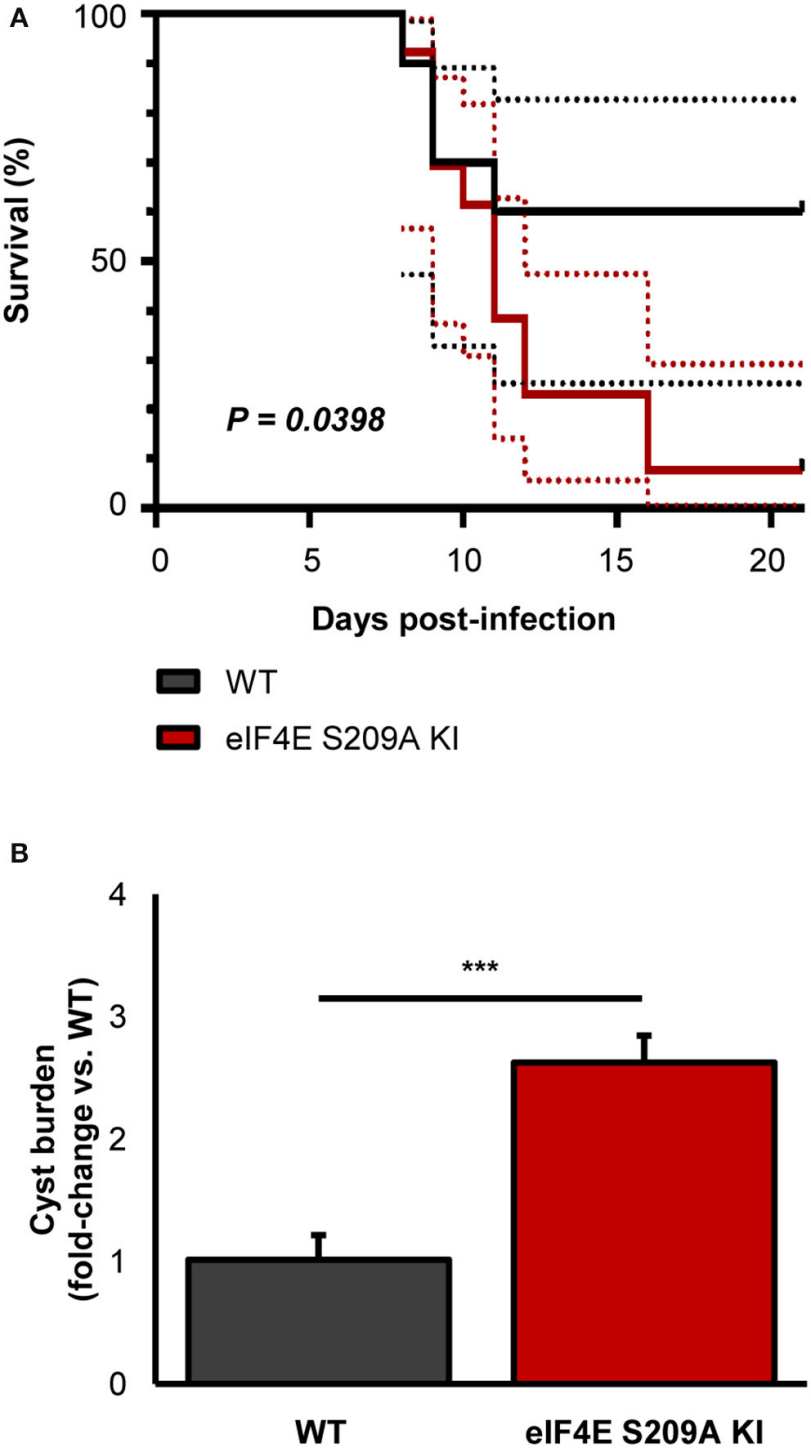
Deficiency in eIF4E phosphorylation confers higher susceptibility to experimental toxoplasmosis and higher brain cyst burdens. Mice were infected IP with 10^3^ ME49 tachyzoites. **(A)** Survival was monitored up to 21 days post-infection. Dashed lines represent 95% confidence intervals. **(B)** Brain cyst burden was measured by qPCR. Results are presented as mean [SD] calculated from values obtained from infected mice (two independent experiments; at least 5 mice per group; all samples were analyzed in technical triplicates. ^***^P < 0.001.

To further extent our *in vitro* observations and to determine the impact of eIF4E phosphorylation on the outcome of toxoplasmosis, we infected and compared WT and eIF4E S209A KI mice. We first measured parasitemia levels in the mesenteric lymph nodes (MLN) and spleens 8 days post-inoculation (i.e., acute phase). Analyses by qPCR revealed a substantial increase in parasite loads in both MLN and spleen tissues in 4E KI mice compared to WT counterparts (Figure 3B). Inflammation appeared to be exacerbated in the former group as revealed by a ~4-fold increase in serum IFNγ concentration (Figure 3C). The observed phenotype in 4E KI mice did not appear to be due to an underlying basal inflammatory state since IFNγ was not detectable in mock-injected animals. In light of these results, we then compared survival rates between 4E KI and WT mice. While most WT animals survived past the acute phase into the chronic phase, 4E KI mice were significantly more susceptible to acute toxoplasmosis with a majority of mortality (~77%) occurring within 2 weeks post-infection (Figure 4A). This heighten susceptibility was reflected by a larger increase in the *T. gondii* cyst burden in the brain of 4E KI mice that survived until the chronic phase of infection in comparison to WT counterparts (Figure 4B). In summary, the absence of eIF4E phosphorylation appears to compromise host resistance against toxoplasmosis despite increased IFNγ production.

## Discussion

Disruption of host signaling pathways and gene expression through altered translation is a strategy employed by diverse pathogens (Mohr and Sonenberg, 2012; Walsh et al., 2013). In this study, our results suggest that *T. gondii* targets and disrupts the host MNK1/2-eIF4E signaling axis, an important translational control node. Both type I and II strains markedly inhibit p38, MNK1/2, and eIF4E phosphorylation in infected macrophages. Interestingly, we have observed a marked difference in the induction of ERK1/2 phosphorylation between the two parasite strains. ERK1/2 phosphorylation increased transiently 1 h after infection by both strains and decreased afterwards. At later time points, however, infection by ME49 led to a gradually increased and sustained phosphorylation of ERK1/2, an observation in line with a recent study that reported a similar trend in bone marrow-derived dendritic cells infected with Prugniaud, another type II strain (Olafsson et al., 2020). Meanwhile, phospho-ERK1/2 levels in RH-infected cells remained low and comparable to basal levels seen in uninfected cultures. Chemical inhibition of phosphatases PP1 and PP2A with okadaic acid restores MNK1/2 and eIF4E phosphorylation but not p38, revealing a disconnection between these signaling mediators following *T. gondii* infection. Seemingly conflicting reports about the regulation of p38 by *T. gondii* are found in the literature. A study by Kim et al. had reported a transient phosphorylation of p38 in BMDMs between 10 to 20 min (RH) and 10 to 60 min (ME49) following infection, after which phospho-p38 levels return to levels comparable to uninfected controls (Kim et al., 2006). It has also been shown that dense granule protein 24 (GRA24) directly interacts with and activates p38 through autophosphorylation (Braun et al., 2013). As the authors of the latter study pointed out, GRA24 presents several alternative splice variants. Thus, under certain growth conditions (e.g., host cell type and/or species, high vs. reduced nutrient availability, time of infection, etc.), *T. gondii* may turn off GRA24-dependent p38 autophosphorylation by synthesizing different GRA24 isoforms (Braun et al., 2013). In contrast, ROP18 is predicted to bind to p38 and cause its degradation (Yang et al., 2017). In our model, we have consistently observed a rapid (i.e., within 15 min after inoculation) and robust inhibition of phosphorylation of p38 by both RH and ME49 strains in both WT and eIF4E S209A KI macrophages. Thus, the reasons for the disparities among the different reports regarding this signaling molecule remain unclear at this point.

The data we obtained with okadaic acid treatment suggest that either parasite-derived phosphatases (devoid of function-altering strain polymorphisms) and/or host phosphatases are implicated in inactivation of the MNK1/2-eIF4E axis by *T. gondii*. To date, the mammalian PP2A phosphatase is the only enzyme shown to dephosphorylate both MNK1/2 and eIF4E (Li et al., 2010). Intriguingly, *T. gondii* GRA16 forms a complex with host PP2A and Herpesvirus-associated ubiquitin-specific protease (HAUSP) (Bougdour et al., 2013). Also, GRA18 binds to host GSK3/PP2A-B56 and to affect β-catenin-mediated gene expression (He et al., 2018). Whether these GRA16- and GRA18-containing complexes display phosphatase activity toward host MNK1/2 and eIF4E or that other host-parasite chimeric complexes are formed remains to be determined. For example, TgWIP, a rhoptry protein, interacts with host SHP2 phosphatase (Sangare et al., 2019). There are 52 predicted phosphatase genes in the *T. gondii* genome of which two encode for the PP2A catalytic subunits, referred to as PP2A1 and PP2A2 (Yang and Arrizabalaga, 2017). The PP2A1 protein sequence contains a signal peptide which raises the possibility that it is secreted into the host cells and targets host MNK1/2 and eIF4E. The fact that STAg failed to recapitulate the effects of live infection does not exclude that soluble factors are linked to MNK1/2-eIF4E dephosphorylation but rather that certain events are required for these factors to mediate their effects within the host cell (e.g., formation and presence of the parasitophorous vacuole membrane, specific route of entry of these molecules, etc.).

The increased replication rate *in vitro*, and parasitemia and virulence in eIF4E S209A KI mice suggest that preventing eIF4E phosphorylation favors *T. gondii* persistence within its host. We observed a substantial increase in serum IFNγ levels 8 days post-infection in 4E KI mice compared to their WT counterparts. Although IFNγ has long been identified as a critical cytokine to control toxoplasmosis (Denkers and Gazzinelli, 1998), it is possible that exacerbated inflammation leads to detrimental effects. Also, it is conceivable that complete absence of eIF4E phosphorylation in infected but also in uninfected 4E KI cells and mice precludes an appropriate immune response to develop against toxoplasmosis. Coincidentally, excessive inflammation has been linked to changes in eIF4E phosphorylation levels in other conditions. In a study by Amorim et al., it was shown that LPS treatment leads to a greater production of inflammatory cytokines IL-2, TNFα, and IFNγ in the brain of 4E KI mice compared to WT animals (Amorim et al., 2018). Similarly, decreased synthesis of the NF-κB inhibitor IκBα has been reported in 4E KI fibroblasts infected with vesicular stomatitis virus (VSV), which leads to increased production of IFNβ (Herdy et al., 2012), and in the brain of 4E KI mice (Aguilar-Valles et al., 2018). Another recent study reported heighten levels of TNFα and IL-1β in 4E KI in old and young mice, respectively (Mody et al., 2020). Thus, higher IFNγ levels in 4E KI mice following *T. gondii* infection could be due to increased parasite burden and/or genetic predisposition to exacerbated inflammation in these mutant mice, which, according to our model, appears to be detrimental to the host. Future studies will be necessary to fully understand the underlying mechanisms linked to the increased susceptibility of 4E KI mice to toxoplasmosis.

It has been reported that phosphorylation of eIF4E regulates translation of a subset of mRNAs including several containing a gamma interferon-activated inhibition of translation (GAIT) element in their 3′ UTR (Amorim et al., 2018), or a 5′-terminal cap and a hairpin structure (Korneeva et al., 2016). It remains to be determined if the translational efficiency of specific subsets of host transcripts is affected by the reduction of phosphorylated eIF4E levels in *T. gondii*-infected cells. Functions of eIF4E beyond translation initiation *per se* are yet to be investigated in the context of parasitic infections. Furthermore, eIF4E-independent effects mediated by MNK1/2 could also play a role during *T. gondii* infection. Other MNK1/2 substrates identified to date include hnRNP A1, PSF, Sprouty2, and cPLA2 (Xie et al., 2019). One study showed that phosphorylation of hnRNP A1 by MNK1/2 decreases its affinity for *Tnfa* mRNA which, in turn, increases translation and synthesis of TNFα by T cells (Buxade et al., 2005). This evidence brings forth the possibility that immune responses mediated by MNK1/2 and its substrates could be dysregulated upon *T. gondii* and warrant future investigation.

In summary, our study identifies the MNK1/2-eIF4E axis as another regulatory node targeted by *T. gondii* to subvert host cell functions and promote its replication. Future studies will allow identification of host- and parasite-derived factors linked to this molecular rewiring and will provide a better understanding of its biological consequences during toxoplasmosis.

## Data Availability Statement

The original contributions presented in the study are included in the article/supplementary material, further inquiries can be directed to the corresponding author/s.

## Ethics Statement

The animal study was reviewed and approved by Comité Institutionnel de Protection des Animaux (CIPA) of the INRS-CAFSB (CIPA 1502-03 and 1611-10).

## Author Contributions

L-PL and MJ conceived and designed the experiments and wrote the manuscript. L-PL, VC, and MJ analyzed the data. L-PL performed the experiments. All authors reviewed and edited the manuscript.

## Conflict of Interest

The authors declare that the research was conducted in the absence of any commercial or financial relationships that could be construed as a potential conflict of interest.
