# Supplementary material for: Infection by the Protozoan Parasite Toxoplasma gondii Inhibits Host MNK1/2-eIF4E Axis to Promote Its Survival
Source: Front Cell Infect Microbiol. 2020 Sep 9;10:488. doi: 10.3389/fcimb.2020.00488 (PMC7509071; doi:10.3389/fcimb.2020.00488)
Supplement: Supplementary file 1 [file Data_Sheet_1.pdf]

Supplementary Information

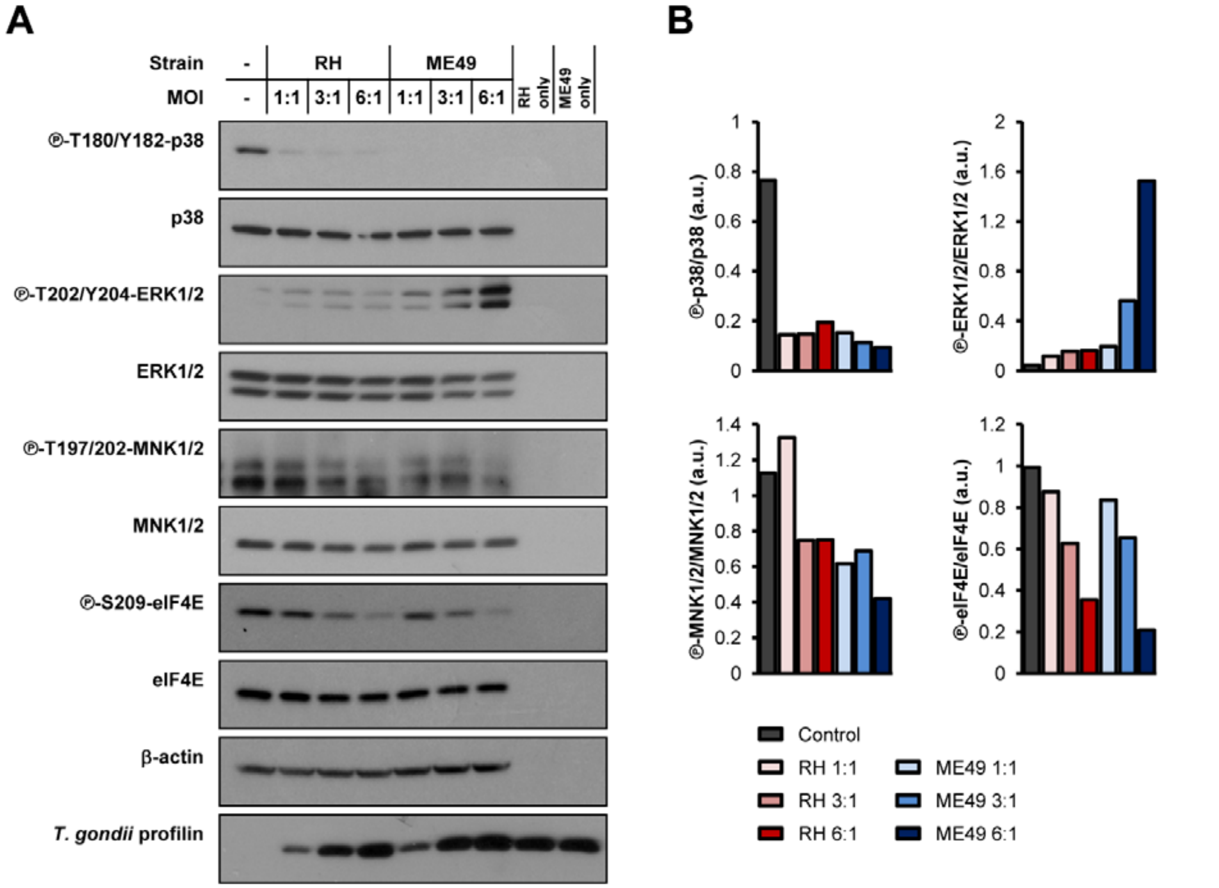

Supplementary Figure 1: Effects of different multiplicity of infection (MOI) ratios on the modulation of MNK1/2-eIF4E signaling axis in macrophages by *T. gondii*. BMDM cultures were inoculated with either RH or ME49 at three different MOIs (1:1, 3:1, and 6:1) for 8 h. (**A**) Phosphorylation and expression levels of indicated proteins were monitored by western blotting. Total amounts of β-actin were used as a loading control and an antibody against *T. gondii* profilin-like protein was employed to assess the infection of the BMDM cultures. Total protein extracts from extracellular tachyzoites (RH and ME49) (*Tg* only) were used to control for any cross-reactivity of the antibodies against *T. gondii* proteins. (**B**) Densitometric analysis of the phosphorylation status of indicated proteins in uninfected control and infected BMDM cultures using FIJI. Data and data analyses are representative of two biological replicates.

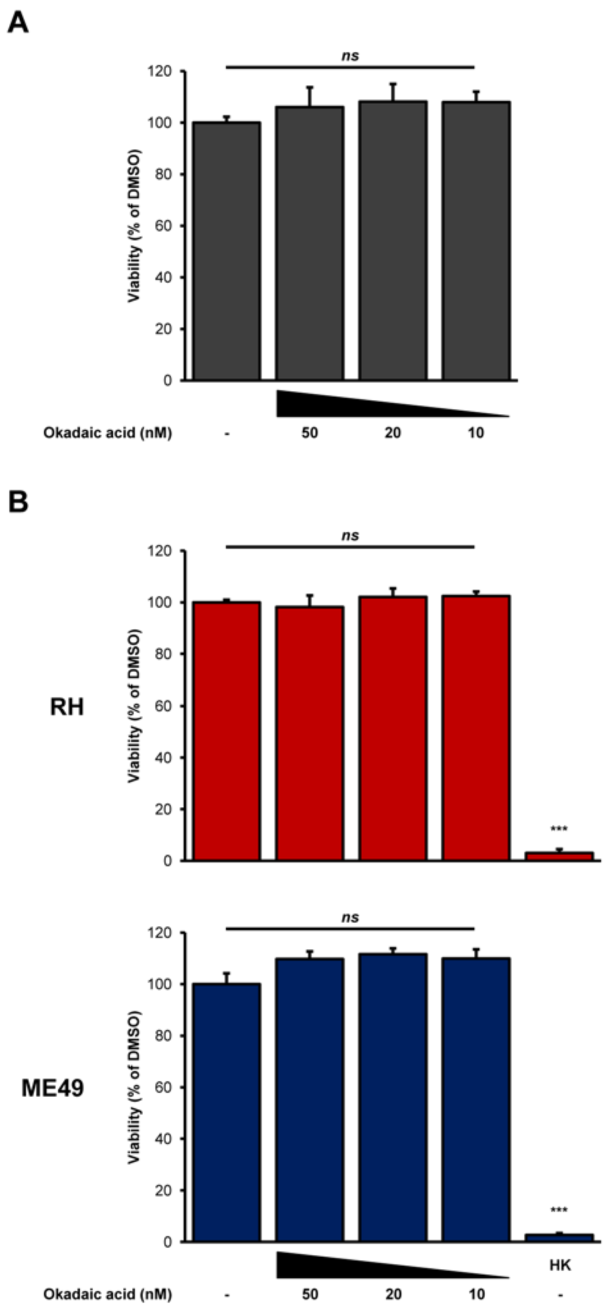

Supplementary Figure 2: Okadaic acid does not affect viability of BMDMs and extracellular *T. gondii* tachyzoites: (**A**) BMDM cultures and (**B**) freshly harvested tachyzoites (devoid of host cells) were treated with increasing doses of okadaic acid or DMSO (vehicle), as indicated, for 8 h at 37°C, 5% CO_2. Resazurin (0.025% final) was added to the culture medium, and (**A**) BMDM were cultured for an additional 4 h while (**B**) parasites were incubated for 16 h.

As a "positive kill" control, heat-killed (HK) parasites (56°C, 10 min) were included. Optical density was measured at 570 and 600 nm, and values were normalized to DMSO-treated samples. Results are presented as mean [SD] and are representative of two biological replicates; all samples were performed in technical triplicates; "*ns*" refers to "not significant".

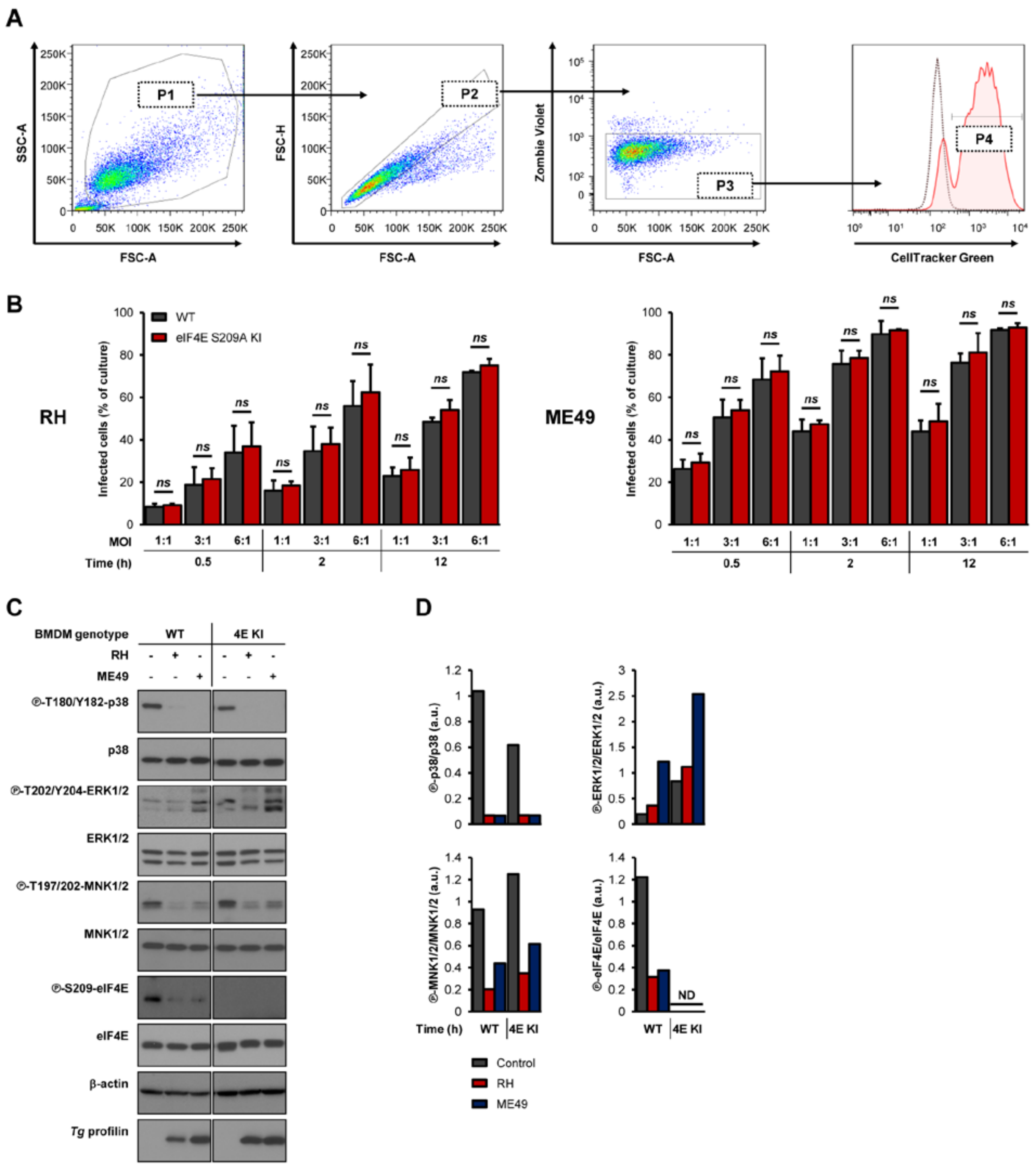

Supplementary Figure 3: Infection rates and modulation of eIF4E upstream kinases by *T. gondii* do not differ between WT and eIF4E S209A KI BMDMs: (**A-B**) WT and eIF4E S209A KI BMDM cultures were inoculated with CellTracker Green-stained RH or ME49 parasites at three different MOIs (1:1, 3:1, and 6:1). Cultures were harvested at the indicated

times, stained with the viability dye Zombie Violet (30 min, RT), then fixed with 1% PFA-PBS (15 min, on ice). Samples were analyzed by flow cytometry using a BD Fortessa. (**A**) Shown here is the gating strategy used to analyze the samples. (**B**) Results are presented as mean [SD] and are representative of two biological replicates; "*ns*" refers to "not significant". (**C-D**) WT and eIF4E S209A KI BMDM cultures were infected with either RH or ME49 (6:1) for 8 h or left uninfected. (**C**) Phosphorylation and expression levels of indicated proteins were monitored by western blotting. Total amounts of β-actin were used as a loading control and an antibody against *T. gondii* profilin-like protein was employed to assess the infection of the BMDM cultures. (**D**) Densitometric analysis of the phosphorylation status of indicated proteins in uninfected control and infected BMDM cultures using FIJI. Data and data analyses are representative of two biological replicates.
